# Impact of Rotor Material Wear on the Aluminum Refining Process

**DOI:** 10.3390/ma15134425

**Published:** 2022-06-23

**Authors:** Tomáš Prášil, Ladislav Socha, Karel Gryc, Jana Svizelová, Mariola Saternus, Tomasz Merder, Jacek Pieprzyca, Martin Gráf

**Affiliations:** 1 MOTOR JIKOV Slévárna A.S., Kněžskodvorská 2277, 370 04 České Budějovice, Czech Republic; tprasil@mjgroup.cz (T.P.); mgraf@mjsl.cz (M.G.); 2 Faculty of Mechanical Engineering, University of West Bohemia, Univerzitní 2732, 301 00 Plzeň, Czech Republic; 3 Environmental Research Department, Institute of Technology and Business in České Budějovice, Okružní 517/10, 370 01 České Budějovice, Czech Republic; socha@mail.vstecb.cz (L.S.); gryc@mail.vstecb.cz (K.G.); svizelova@mail.vstecb.cz (J.S.); 4 Faculty of Materials Engineering, Silesian University of Technology, Krasinskiego 8, 40-019 Katowice, Poland; tomasz.merder@polsl.pl (T.M.); jacek.pieprzyca@polsl.pl (J.P.)

**Keywords:** aluminum refining, graphite wear, wear testing, rotary impeller

## Abstract

The paper presents the results of tests carried out during the refining of the AlSi9Cu3(Fe) alloy in industrial conditions at the FDU stand. In the tests, three different rotors made of classical graphite, fine-grained graphite and classical graphite with SiC spraying were tested for the degree of wear. A series of tests was conducted for five cases—0% to 100% of consumption every 25%—corresponding to the cycles of the refining process. The number of cycles corresponding to 100% wear of each rotor was determined as 1112. The results of the rotor wear profile for all types of graphite after the assumed cycles are presented. Comparison of CAD models of new rotors and 3D scans of rotors in the final stage of operation revealed material losses during operational tests. The study assessed the efficiency of the rotor in terms of its service life as well as work efficiency. It was estimated on the basis of the calculated values of the Dichte Index (DI) and the density of the samples solidified in the vacuum. The structure of samples before and after refining at various stages of rotor wear is also presented, and the results are discussed.

## 1. Introduction

Currently, one of the stages of aluminum production is refining, which allows the removal of metallic and non-metallic impurities from the liquid metal, including hydrogen, which causes porosity and thus deteriorates the mechanical properties of the material [1,2,3]. In recent years, there has been a continuous development of techniques for introducing refining gas in the production process of high-grade aluminum. Starting with a single lance, a pile of lances with additional equipment, porous plugs, usually ended with rotors with graphite blades [4,5,6]. Therefore, nowadays, the refining process is very often carried out in reactors with rotors through which refining gas is introduced, most often argon or a mixture of argon and chlorine. The essence of the process is the introduction of fine gas bubbles into the liquid metal, which simultaneously mix the metal and remove unnecessary gaseous and non-metallic impurities as a result of flotation [7,8,9].

In recent years, the topic of aluminum refining has attracted many researchers. Most of the works focus on understanding the phenomena occurring during the process, which is possible thanks to the use of physical modeling [10,11,12,13,14,15]. More and more often, this modeling is supplemented and at the same time verified by numerical modeling [16,17,18,19,20]. However, many studies are still based on experiments conducted directly under industrial conditions [21,22,23,24,25]. An important topic is the durability of the rotor material. Various types of graphite are used as the material for the impeller and rotor shafts due to the ease of machining and non-wettability with metals. It is not without reason that graphite is used for elements of machines and rotors, because it is resistant to the refining gases used, i.e., chlorine, nitrogen and argon, as well as their mixtures and other aggregates. After immersion in a liquid metal bath, an element made of graphite is subjected to a rapid (fractional) temperature change from room temperature to several hundred degrees Celsius. At the same time, the rotor is exposed to cold refinery gases. Moreover, the very use of a rotor requires a high degree of strength, since the rotating gas distribution rotor heads are immersed in the melt during rotation. Therefore, one of the disadvantages of graphite as a material for rotors is its oxidation and erosion. Therefore, the paper presents test results for the same type of rotor with an impeller made of three different types of graphite. This type of research is unique, but many researchers are increasingly focusing on topics related to the durability and consumption of materials used in industry, although this type of research is not easy [26,27,28].

## 2. Research Study and the Object

The refining process was carried out on the AlSi9Cu3(Fe) alloy using an FDU unit with a rotor. The AlSi9Cu3(Fe) alloy was obtained from secondary materials (scrap); therefore, after each series of experiments, the chemical composition was determined with using optical emission spectrometry—the average chemical composition within the standards is presented in Table 1.

**Figure 1:**
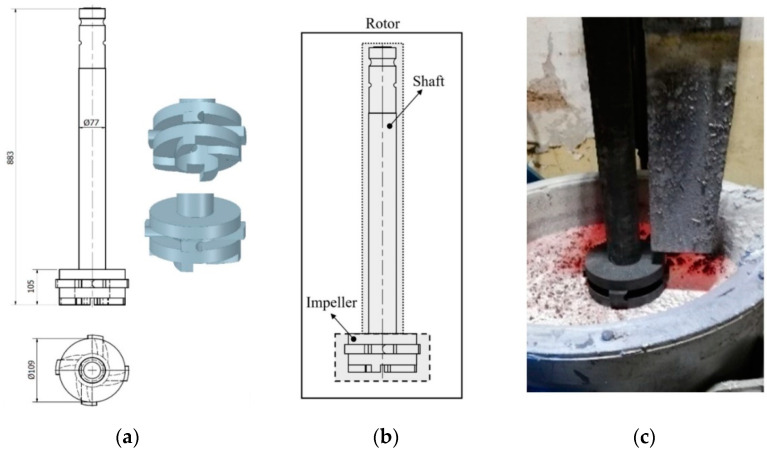
View of: (**a**) Dimensions of tested rotor (mm); (**b**) Description of tested rotor; (**c**) Use of rotor in operational conditions.

The research focused on the assessment of the refining efficiency at the FDU stand, as shown in Figure 1a, with different degrees of wear of various types of graphite rotor, the scheme of which is shown in Figure 1b with the characteristic dimensions marked. Table 2 presents the processing parameters; additionally, three types of tested rotors, which differed in the material from which the rotor was made, are marked in it.

In the first case, it was classic graphite, in the second, it was fine-grained graphite, and in the third, it was classic graphite with SiC spraying. Table 3 presents the basic parameters of these rotors, such as density, flexural strength and porosity. Rotors A1 and A2 are additionally impregnated with refractory material. This impregnation is to prevent the oxidation of the graphite, which should extend the service life of the FDU rotor. In the case of the A3 rotor, SiC refractory spraying was used instead of impregnation. The spray was applied to the entire surface of the shaft and impeller. Spraying is to prevent abrasive wear and oxidation of the graphite, which can extend the life of the FDU impeller.

The course of refining and the life of the rotors were assessed on the basis of the number of cycles at which a certain percentage of rotor wear is achieved. The achievement of individual stages or a specific degree of rotor wear was determined after consultation with the suppliers of individual rotors together with one of the local die-casting foundry company (for automotive), based on experience and internal regulations. Therefore, as part of the experiments, a series of tests was carried out for five cases (0% to 100% of consumption every 25%) corresponding to the cycles of the refining process. One cycle is 3 min of rotor operation. The wear values assigned to the respective cycles are summarized in Table 4. The number of cycles corresponding to 100% wear of each rotor was determined by the manufacturer as 1,200. The number of cycles corresponding to the individual stages of rotor wear was derived from this value. However, due to the production possibilities, it was not feasible to adhere exactly to the specified number of cycles. Due to the loss of A1 rotor material and the operational safety, the tests were completed before 1200 cycles. Therefore, 100% wear of the A1 rotor corresponds to 1112 cycles.

All samples of experiments series 1/VT to 5/VT (0% to 100% wear) were selected to give an idea of the course and nature of refining efficiency. Selected series of experiments represent 144 melts, where we focused on the efficiency of melt refining during production within the following technological flow: AlSi9Cu3(Fe) alloy → STRIKO shaft furnace → FDU → UP maintenance furnace → die casting → ROCKER COVER.

In order to assess the efficiency of refining, alloy samples for the density index test were taken before (FDUs) and after refining (FDUe)—it gives possibility to evaluate visual stages of degassing—Dichte Index test (80 mbar).

Gasification is usually referred to as double weighing. The principle of the method is to compare the density of an alloy sample solidifying under atmospheric pressure with a sample solidifying under reduced pressure. The Sieverts law is applied here, according to which the solubility of hydrogen in the metal is lower under reduced pressure, and thus, more hydrogen bubbles are removed during solidification than at atmospheric pressure. The bubbles remain enclosed in the metal and thus reduce its density. A sample of the alloy to be assessed is taken from the melting furnace and poured into two test crucibles (metal, ceramic or sand mix—according to the manufacturer) with a volume of about 40–80 mL. One sample is allowed to solidify at atmospheric pressure, while the other is allowed to solidify under a defined vacuum in a vacuum chamber. The density of the sample solidified under atmospheric pressure *ρ_s atm_* and the sample solidified in vacuum *ρ_s vak_* is determined by weighing in air and after immersion in a beaker with water, using the principle of Archimedes’ law. The sample densities are calculated according to the general formula:

(1) $$\rho_{s}=\frac{m_{s}}{V_{s}}=\frac{m_{s}}{m_{s\;H2O}}.\rho_{H_{2}O},$$

From the densities *ρ_s atm_* and *ρ_s vak_*, the density index known as Dichte Index (DI) is determined according to the formula:

(2) $$\mathrm{DI}=\frac{\left(\rho_{s\;atm-}\rho_{s\;vak}\right)}{\rho_{s\;atm}}\cdot 100\%,$$

The double weighing method is probably the most common and relatively accurate method in foundries today. The value of the DI in conventional castings is usually around a few percent. Modern instruments designed for this method only require weighing of the sample in air and immersed in water. The density is calculated automatically. The DI expresses the overall effect of the content of gases and oxide inclusions—that is, the actual tendency of an alloy to form bubbles.

## 3. Research Results and Discussion

Table 5 shows the results of rotor wear profile for all graphite types after the cycles listed in Table 4. The wear of the A1 rotor after 25% of refining cycles is not high. Droplets of material appear on the rotor shaft but also on the impeller, but no reduction in the rotor material has been observed. After 50% of refining cycles, the dripping of the material is significantly greater, and the lower edge of the impeller has also decreased. After 75% of refining cycles, the material drips are significant, and the lower edge of the impeller has decreased significantly from the baseline. Throughout 100% of the refining cycles, the rotor is still operational, but the bottom edge is very negligible. Significant material infiltration was also observed on the shaft and baffle as well as on the impeller.

The wear of the A2 rotor after 25% of refining cycles is high compared to the A1rotor. Significant infiltration on the shaft and the impeller have been found; moreover, the lower edge of the impeller is significantly damaged and worn. After 50% of refining cycles, the rotor wear is close to 25% of refining cycles; only a reduction in the lower edge of the impeller was observed. After 75% of refining cycles, the material wear is significant, a significant material drip on the shaft and the rotor itself has been observed, and the lower edge of the impeller is already significantly worn. Unfortunately, the rotor did not survive the 100% refining cycles.

In the case of the A3 rotor, after 25% of refining cycles, a very high wear of the rotor material was observed, and the lower edge of the rotor is very worn (very thin), which proves its fast wear. The rotor made of this material did not withstand up to 50% of the refining cycles. This material was worn much faster than the A1 and even the A2 rotors.

**Figure 2:**
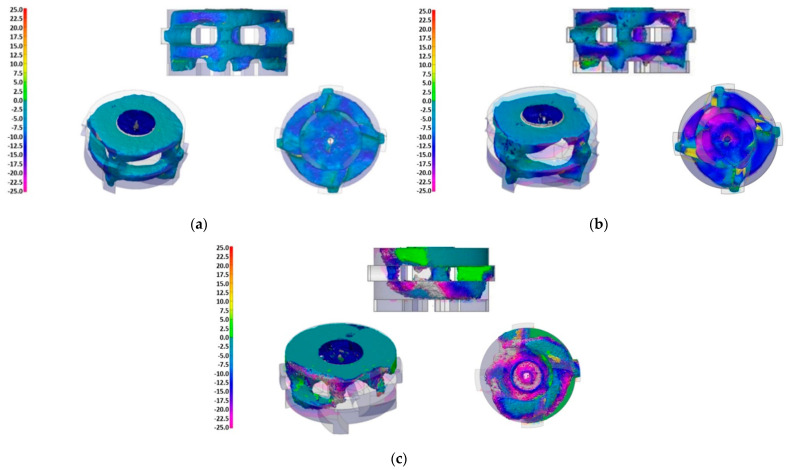
Rotor wear, 3D scanner: (**a**) Material loss 36.9%, Impeller A1; (**b**) Material loss 45.7%, Impeller A2; (**c**) Material loss 28.8%, Impeller A3.

A comparison of the CAD models of new rotors and 3D scans of the rotors in the final stage of operation revealed material losses during operational tests. The dimensional deviations from the original shape of the rotors are shown in Figure 2. The rotor A1 shows a material loss of 36.9%. The maximum dimensional deviations of the rotor A1 reached the values of about 10 mm and are located on the underside of the impeller. Rotor A2 shows a material loss of 45.7%. The maximum deviations were around 25 mm and mainly occurred in the area of the blade recesses on the underside of the impeller. In the case of the A3 rotor, the material loss reached 28.8%. The maximum deviations from the original shape were about 25 mm and occurred in the area of the blade recess on one side of the impeller.

**Figure 3:**
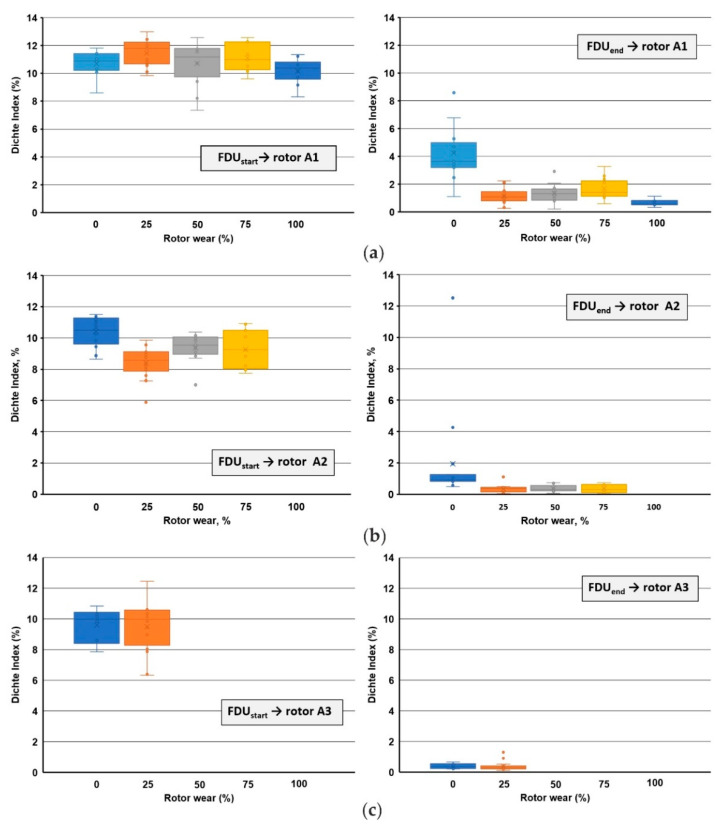
Comparison of Dichte Index values within rotors wear: (**a**) rotor A1 (series 1/VT to 5/VT); (**b**) rotor A2 (series 1/VT to 4/VT); (**c**) rotor A3 (series 1/VT to 2/VT).

The Dichte Index (DI) values, as well as the density of samples solidified in vacuum, were determined from the results of the vacuum test of individual melts. Within the results, a comparison of the values of the Dichte Index (DI) at the beginning of the refining process (FDUstart) and at the end of the process (FDUend) is presented in Figure 3 for rotors A1, A2 and A3.

The achieved results of the Dichte Index (DI) show that before the refining process, the melt shows an increased gas content for A1 rotor $\bar{\mathrm{DI}}=10.68\%$ and $\bar{\mathrm{DI}}=10.16\%$ for both series 1/VT and 5/VT (0% and 100% wear; Figure 3a). As part of the refining process carried out in the FDU device, we observed a significant reduction in DI, which is evident from the achieved values: $\bar{\mathrm{DI}}=4.25\%$ and $\bar{\mathrm{DI}}=0.67\%$, for both series 1/VT and 5/VT (0% and 100% wear; Figure 3b). A similar behavior of the Dichte Index is observed for the A2 and A3 rotors. For the A2 rotor Dichte Index at the beginning of the refining, the following level is observed: $\bar{\mathrm{DI}}=10.41\%$ and $\bar{\mathrm{DI}}=9.26\%$ for series 1/VT and 4/VT (0% and 75% wear). At the end of refining, the Dichte Index values were the following: $\bar{\mathrm{DI}}=1.94\%$ and $\bar{\mathrm{DI}}=0.37\%$ for series 1/VT and 4/VT (0% and 75% wear). Whereas for the A3 rotor, the Dichte Index at the beginning of the refining was: $\bar{\mathrm{DI}}=9.60\%$ and $\bar{\mathrm{DI}}=9.51\%$ for series 1/VT and 2/VT (0% and 25% wear), and at the end of refining: $\bar{\mathrm{DI}}=0.39\%$ and $\bar{\mathrm{DI}}=0.38\%$ for series 1/VT and 2/VT (0% and 25% wear).

**Figure 4:**
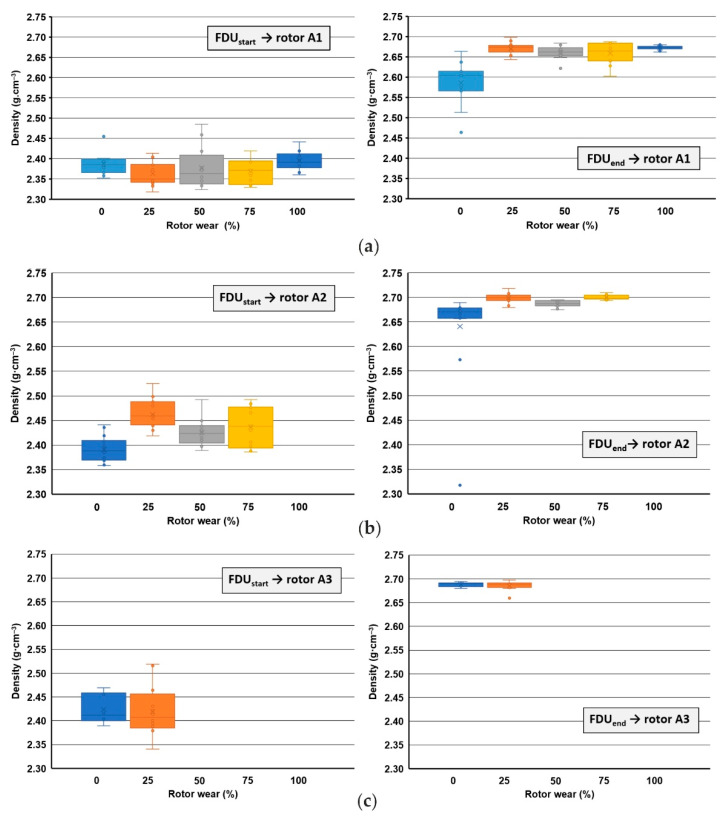
Comparison of densities values of samples solidified in vacuum within the rotors wear: (**a**) rotor A1 (series 1/VT to 5/VT); (**b**) rotor A2 (series 1/VT to 4/VT); (**c**) rotor A3 (series 1/VT to 2/VT).

The results of the DI after the FDU treatment show a significant difference between the rotor showing 0% and 100% wear. With 0% wear of the rotor (series 1/VT), it can be concluded that the rotor contains moisture which is gradually released. With a 100% rotor wear (series 5/VT), it can be stated that even with such a high wear, the rotor achieves the lowest DI not only within the average values but also within the individual melts. These trends for DI are also seen in the density results of vacuum-solidified samples. Figure 4 presents a summary of changes in the alloy density for the beginning and end of refining for the three tested rotors A1, A2 and A3.

**Figure 5:**
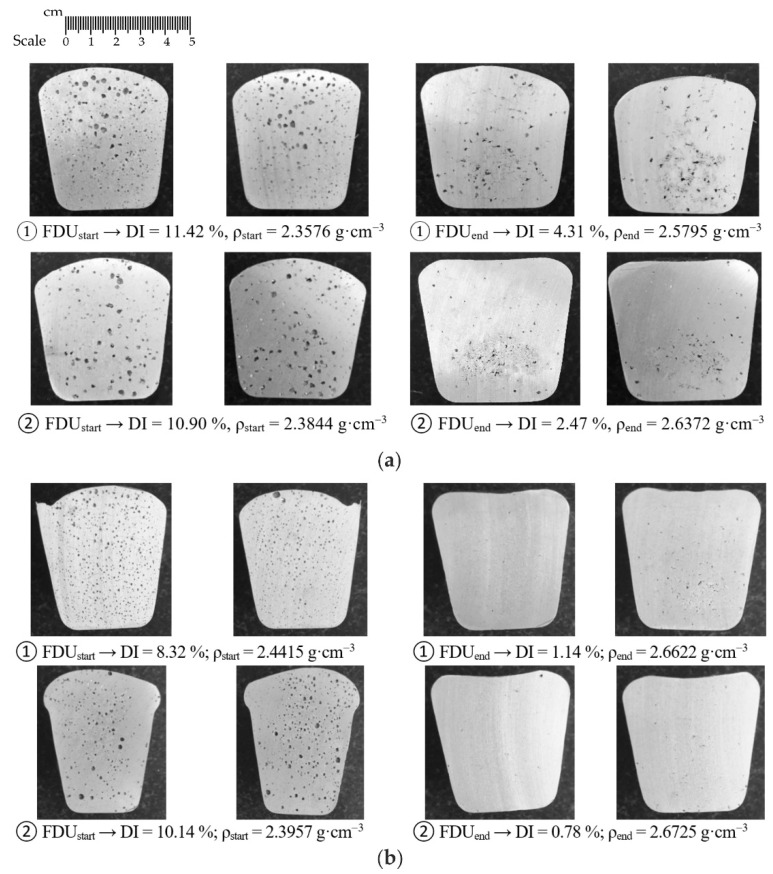
Sections of samples taken for gasification assessment for rotor A1: (**a**) wear 0% (series 1/VT); (**b**) wear 100% (series 5/VT).

**Figure 6:**
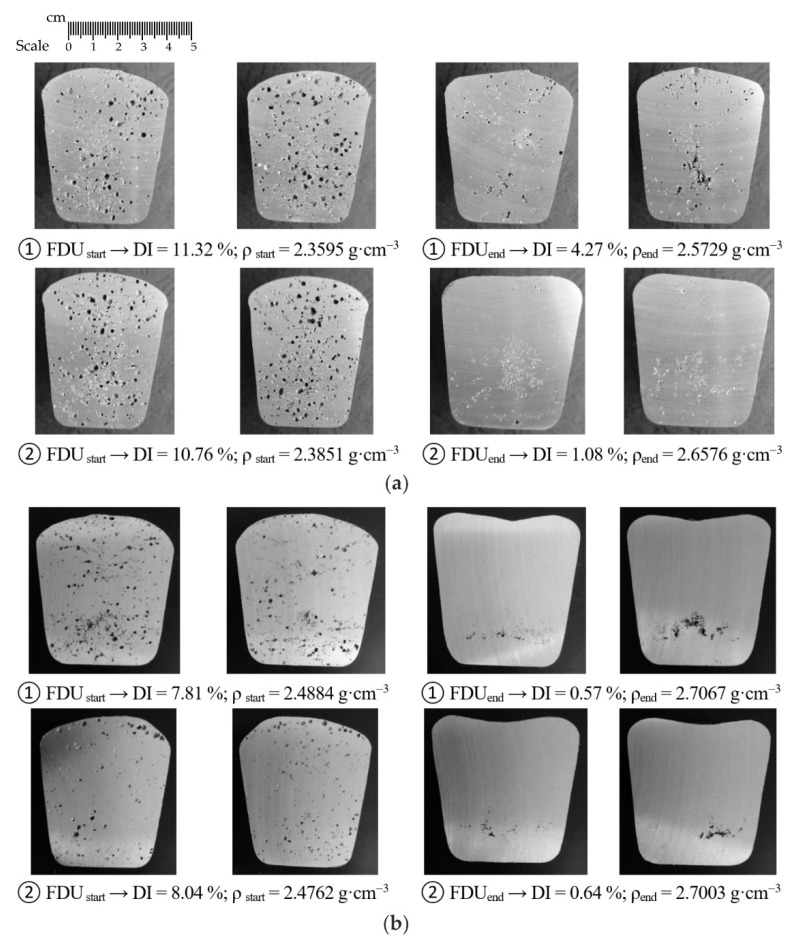
Sections of samples taken for gasification assessment for rotor A: (**a**) wear 0% (series 1/VT); (**b**) wear 75% (series 4 /VT).

**Figure 7:**
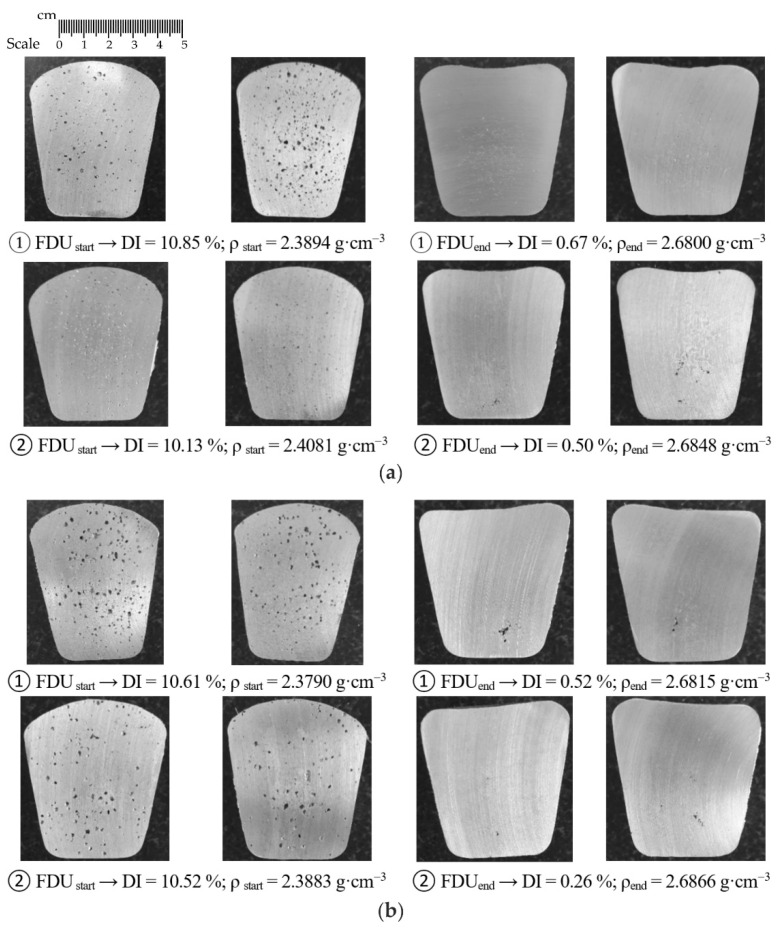
Sections of samples taken for gasification assessment for rotor A3: (**a**) wear 0% (series 1/VT); (**b**) wear 25% (series 2/VT).

In addition to determining the Dichte Index (DI) and the density of the vacuum solidified samples, the evaluation of the vacuum solidification tests was performed by visual cross-section evaluation. The most interesting series of heats were selected for testing, which always contained samples from the two heats that had the largest initial DI difference. For series 1/VT (rotor wear 0%) and 5/VT (rotor wear 100%), images of individual samples for the Dichte Index for rotor A1 are shown in Figure 5; whereas for rotors A2 and A3, respectively, these images are shown in Figure 6 and Figure 7. The sampling point and the achieved Dichte Index are indicated in each image.

In the case of rotor A1, at the beginning of the refining process, significant porosity is observed in the entire sample volume. At the end of the refining cycle, the porosity is much less, but some gas bubble residues are still visible in some places in the sample (see Figure 5a). Much better results are observed for 100% rotor wear, especially after completion of the refining cycle. There is practically no porosity in the sample volume; perhaps only a few tiny residual gas bubbles can be seen in the selected samples (see Figure 5b). Similar conclusions can be applied to rotors A2 and A3.

**Figure 8:**
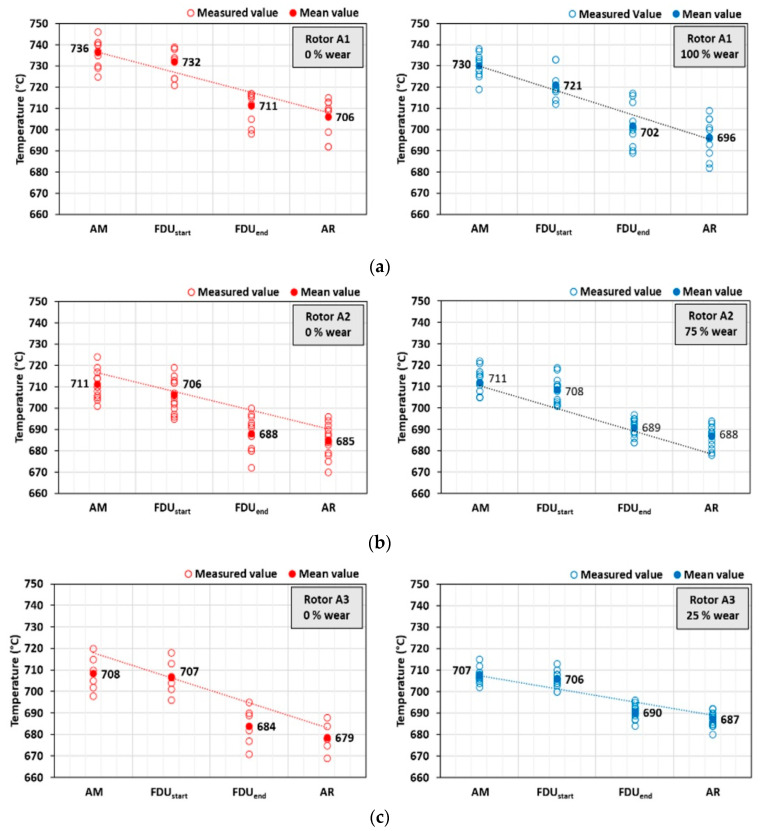
AlSi9Cu3(Fe) melt temperature drop during production (from melting to refining): (**a**) rotor A1 (series 1/VT and 5/VT), (**b**) rotor A2 (series 1/VT and 4/VT), (**c**) rotor A3 (series 1/VT and 2/VT).

As part of individual series of experiments, the melting point was also measured at the following technological sites: AM (After Melting), FDU_start_, FDU_end_ and AR (After Refining). An evolution of temperature drop during die casting for each rotor is summarized in Figure 8. The achieved temperature drops show the same trend, including places with the greatest heat loss, during processing at the FDU unit, when the melt temperature decreases by an average of 15.5 °C to 23.4 °C. The total temperature drop of AlSi9Cu3(Fe) during processing before casting into the holding furnace was 25.3 to 33.6 °C.

## 4. Conclusions

Based on the research, the following conclusions were drawn:

− There is a certain amount of moisture in the new rotor, which contributes to re-gasification; as the rotor wear increases, the degree of degassing of the metal bath increases, and thus, the porosity of the obtained cast samples decreases;
− For the A1 impeller made of classic graphite impregnated with refractory material, the longest service life was observed: about 1100 refining cycles;
− The A2 impeller, made of fine-grained graphite, also impregnated with refractory material, has a slightly shorter service life than the A1 impeller; the impeller survived about 900 refining cycles;
− The worst impeller in terms of service life turned out to be the A3 impeller (SiC sprayed graphite); its wear is observed after less than 350 refining cycles, which means that there is no recommendation for further use.

The obtained results allowed mapping the efficiency of refining/degassing of molten AlSi9Cu3(Fe) during technological treatment with 0% to 100% wear of various types of graphite rotors. Based on the above results, it will be possible to compare the efficiency of refining using a different type of rotor to the die casting process.

## Figures and Tables

**Table 1:** Average chemical composition of AlSi9Cu3(Fe) alloy samples taken during operational tests.

Chemical Composition, wt %											
Si	Fe	Cu	Mn	Mg	Cr	Ni	Zn	Pb	Sn	Ti	Al
8.551	0.943	2.301	0.312	0.202	0.063	0.066	0.930	0.057	0.021	0.041	86.48

**Table 2:** Refining process parameters.

Parameter of Refining Process		Unit
Refining time	180	s
Rotary impeller speed	350	rpm
Flow rate of gas	17	dm^3^⋅min^−1^
Working height	200	mm

**Table 3:** Impeller designation and characterization of material used for rotor preparation.

Designation	Characteristics of Materials			Parameters of Graphite Materials		
	Graphite Type	Impregnation	SiC Spraying	Density, g·cm^−3^	Flexural Strength, MPa	Porosity, %
A1	Classic graphite	Yes	No	1.67–1.74	12.0–16.6	20.0–24.0
A2	Fine-grained graphite	Yes	No	1.72–1.75	15.5–19.5	15.0
A3	Classic graphite	No	Yes	1.67–1.74	12.0–16.6	20.0–24.0

**Table 4:** Course of operational tests and rotor wear values for all rotors.

Experiment Series	Impeller Wear	The Beginnings of the Cycle		
		A1	A2	A3
1/VT	0%	0×	0×	0×
2/VT	25%	287×	366×	343×
3/VT	50%	500×	607×	
4/VT	75%	798×	875×	
5/VT	100%	1112×		

**Table 5:** Comparison of rotors wear profile at different stages of service life.

Percentage Level of Rotor Wear, %				
0%	25%	50%	75%	100%
Rotor A1				
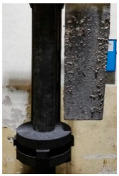	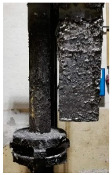	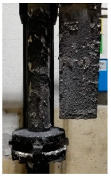	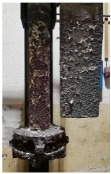	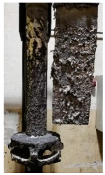
Rotor A2				
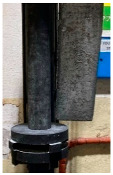	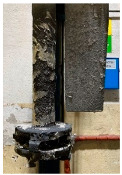	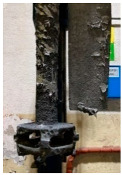	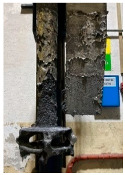	-
Rotor A3				
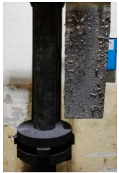	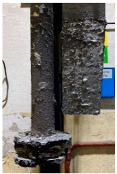	-	-	-

## Data Availability

Not applicable.
